# Book Review

**Published:** 1893-12

**Authors:** 


*The Diagnosis of Intra-ocular Growths.* By A. Hill Griffith, M.D. Pp. 24. London: H. K. Lewis. 1892.—This pamphlet contains an account of many eyeballs examined by the author after enucleation, and his comments thereon. Dr. Hill Griffith has paid special attention to the subject, and his publication is worthy of careful perusal. Under glioma, he notices the rare conditions non-vascular glioma and glioma endophytum. He then deals with sarcoma of the choroid, and points out the difficulty of a certain diagnosis in the first stage from retinal detachment, particularly where serous exudation intervenes between the tumour and the overlying retina; and in the second stage, from acute glaucoma. Several cases of cysticercus are also mentioned.

*Scab-Healing and its Application in General Surgery.* By J. Delpratt Harris. Pp. vi., 28. London: H. K. Lewis. 1893.—Under the above heading the author describes his method of dressing wounds with sawdust pads, which have proved highly effectual in his hands. He does not seem to distinguish between *aseptic* and *antiseptic* surgery, and his pathology is rather old-fashioned; but the pamphlet is well worthy of perusal, as showing what can be done with simple means

*Burdett's Hospital Annual and Year-Book of Philanthropy.* London: The Scientific Press Limited. 1893.—The fourth volume of this excellent work is deserving of all praise, for the immense amount of information it contains, for its arrangement, and for the way in which it is printed. Everyone who is engaged or interested in hospital management should possess this book; and Mr. Burdett is glad to receive information, so as to make it as accurate and complete as possible. There is a most exhaustive list of hospitals, dispensaries, lunatic asylums, convalescent homes, nursing institutions, orphanages, &c., in London, in the provinces, in Scotland and Ireland, in the Colonies, and in America. The number of beds, of patients relieved, the expenditure and income, with the conditions of relief and other information, are stated for each of the charities. There are also chapters on such cognate subjects as "Character and Cost of Work Done in our Philanthropic Institutions," "The Income of Voluntary Hospitals," "Hospital Sunday and Saturday," "Nurses and Nursing," &c. Mr. Burdett is a recognised authority; he deals with these matters in a statesmanlike spirit. His book gives an easy opportunity for comparing notes among hospital managers, and for arriving at a general improvement in the administration of medical charity. Local matters are not given quite accurately. The Bristol Royal Infirmary is placed in the list of Provincial Hospitals with Medical Schools, but the Bristol General Hospital in that of Provincial Hospitals without Schools. The Infirmary and Hospital have the same relation to the medical school, which is now the Faculty of Medicine in Bristol University College. Although the volume is dated 1893, some of the statistics have an ancient look, as in most of the cases the figures are for the year 1891.

*Year-book of the Scientific and Learned Societies of Great Britain and Ireland.* London: Charles Griffin & Company Limited. 1893.—This useful work answers the purposes for which it was compiled; viz., to give information about the various scientific *and* (there is hidden satire in this "and") learned societies which abound. Their days of meeting, their presidents and secretaries, and in some instances the work done by them during the previous year, can easily be ascertained. It is, no doubt, difficult for the compilers always to obtain the knowledge they require; but we are rather surprised to see only a line and a-half of small print devoted to the Bristol Medico-Chirurgical Society, whereas more than half a page of bold type is given to a club whose records contain papers on "Baby Beef," "The Welsh Pony," and "Cheddar Cheese."

*The Indian Medico-Chirurgical Review.* Bombay: Rao & Co.—We are glad to welcome this new monthly as an exchange. Judging by the varied contents of the early numbers, we believe that the energy of Dr. Choksy and his coadjutors will succeed in providing the western side of India with a trustworthy and scientific monthly medical magazine.

*The Post-Graduate.* New York.—This monthly journal is another addition to our exchange list. It consists mostly of papers read in the Clinical Society of the New York Post-Graduate Medical School, with discussions thereon. The material is always interesting, and is frequently well illustrated.

*Saint Bartholomew's Hospital Reports.* Vol. XXVIII. London: Smith, Elder & Co. 1892.—In compiling the report for the year 1892, the editors have followed the lines laid down in the previous volumes of not confining their articles to the experiences of their own hospital, and have admitted papers which we presume are by old Bartholomew's students. In the present volume there are numerous papers of value. While there are none which contain any very original ideas, there are some of great interest. Mr. Howard Marsh relates some cases to show the difficulties in arriving at a correct diagnosis between new growths and inflammatory enlargements of bones. Mr. Harrison Cripps lays down directions for the incisions for abdominal section. Should difficulty be found in reducing a distended gut, he recommends a small incision in the wall of the intestine, kneading out the fluid and gaseous contents, and closing the wound with Lembert's sutures. Dr. Haig discusses the probability of the presence of excess of uric acid acting as the chief cause of Raynaud's disease. A melancholy interest attaches itself to the last paper—an excellent one on "Typhoid Fever," by Dr. Tylden,—as it was found incomplete after the death of that promising physician from that very disease. The volume is up to the usual high standard of the Saint Bartholomew's Reports. It will be found to contain many other papers of importance. It also contains a list of specimens added to the museum during the year and the medical and surgical report.

*The Leeward Islands Medical Journal.* Vol. II. London: J. & A. Churchill. 1892. — Truly the British Medical Association is an institution possessing a wonderful vitality. This is evidenced by the manner in which its various branches have sprung up all over the kingdom and in some very remote corners of our colonial empire. Here is the second volume of the journal of a young branch, consisting of medical men practising in eight of the Lesser Antilles. Their opportunities of meeting are difficult and precarious. There is not much inter-communication between the smaller islands of the West Indies. There are few coasting steam vessels, and very often small sailing vessels afford the only means by which residents of one island can visit those of another. We are glad to observe the encouragement given to them by the local government, which undertakes the publication of the proceedings. In this volume the papers on "The Bilious Remittent Fever of the Tropics" are especially good. The book is well printed, and altogether reflects much credit on the officers of the branch. We heartily wish the effort every success.

*Modern Homeopathy: Its Absurdities and Inconsistencies.* By William W. Browning, M.D. Pp. 32. Philadelphia: Wm. J. Dornan. 1893.—The homeopathic heresy dies hard. This pamphlet has been forwarded to us by Dr. Geo. M. Gould, of Philadelphia, whose prize of 100 dollars was awarded to the essay, designed for distribution by physicians in order to disseminate more enlightened views upon the subject. The enthusiasts who are disposed to accept what they do not understand would do well to give an attentive perusal to this clear and well-written account of the Hahnemannian system. The reasons why the medical profession reject its pretensions are made abundantly clear. The so-called claims of Homeopathy should not be contemptuously rejected without full investigation, but little of the experimental method will be needful. Dr. Gould's contributions to Medicine are many and various. It is a good service to have been the means of producing such an essay as this. He is well known as the editor of the *Medical News* and the author of a compact and useful medical dictionary, and we hear that he now has almost ready a new illustrated dictionary of medicine, biology, and the collateral sciences, upon which he and many assistants have been engaged for several years.

*From Hospital-Ward to Consulting-Room: A Medical Autobiography.* By a Graduate of the London University. Pp. 58. London: H. K. Lewis. 1893.—This is a plain, unpretending story of the pursuit of knowledge under difficulties. It "describes the career of a medical student who, by dint of industry and perseverance, made his way to the highest place in his profession," by which he means the degree of M.D. of the University of London. Whether or not he overrates his Alma Mater may be a question; but we hope that no ill-advised attempts to render this degree more easily obtained will end in impairing its justly earned prestige. The principal interest of this book consists in the narrative of the difficulties which the writer, who was born in a humble position, had to overcome in order to obtain this much-coveted degree. One of the large class who have most of the voting power, who consider themselves as *par excellence* the "working-men" of the country, and who go in for eight hours bills, would probably stand aghast at his record of hard work and self-denial. The young medical man who possesses ability and, above all, industry, and who cannot afford to wait for practice, will find this little book full of encouragement to him in the uphill work which is to be the business of his life. It abounds in shrewd remarks and sensible observations upon the daily life and trials of the general practitioner.

*Recreation.* By William Odell. Pp. 44. Torquay: A. Iredale. 1893.—"The compatibility of excellence in mental and physical exercise" would have been, in our opinion, a more fitting title for this address, which was read before the Torquay Natural History Society. It is generally, if not universally, admitted that a certain amount of relaxation from one's ordinary vocation is not only beneficial, but necessary; and this relaxation, as a rule, takes the form of muscular exercise combined with skill. But when the question is raised whether it is possible to excel in brain work and physical recreation, we have another matter under consideration. Mr. Odell appears to us not to sufficiently recognise the difference between these two questions, and forgets that too much play will make as dull a boy of Jack as too much work. We can all call to mind examples of bishops, judges, and other men eminent in their professions and callings who have in their younger days figured prominently on the cricket-field, the cinder-path, or the river; but there are a larger number of men, we believe, who have failed in the Schools, or, at any rate, not taken a high place, because their athletic pursuits interfered with their studies. There are no records of them: they are forgotten or overlooked. They could not serve two masters, and, perhaps, naturally chose the one who offered the most attractions. The various opinions of the head-masters collected by Mr. Odell are interesting from their diversity. No one would have believed that men placed in almost similar positions, and with like opportunities of observation, could have come to such opposite conclusions. We are, however, inclined to to agree with the Provost of Eton, who says that "as games become more scientific, as they undoubtedly do, the number of men who combine success in school and university with high athletic proficiency will be found to grow smaller and smaller." Mr. Odell expresses no opinions of his own.

*Obstetric Aphorisms.* By JOSEPH GRIFFITHS SWAYNE, M.D. Tenth Edition. London: J. & A. CHURCHILL. 1893.—For many a year Dr. Swayne's well-known *Aphorisms* have been a comfort and strength to countless obstetric neophytes. The fact that a tenth edition has just been published is ample testimony to the worth of the book, which is kept up to date. The new issue is distinguished by an appendix on aseptic and antiseptic midwifery.

*Annual of the Universal Medical Sciences.* Edited by CHARLES E. SAJOUS, M.D. Five volumes. Philadelphia: The F. A. DAVIS COMPANY. 1893.—The world is much indebted to the editor and his seventy colleagues for the painstaking care with which this, the sixth issue of the colossal *Annual*, has been duly consummated. A prolonged sojourn in Europe has enabled the editor to widen the scope of the work, and to increase its sphere of usefulness by giving it more of an international character. He not unnaturally extols the American element, and points out that many really valuable articles published in the United States would be unknown on the continent of Europe but for the assistance of this *Annual*. "It were vain to deny their sufficient worth. Many neglected works are, indeed, far above the average of general medical literature. As regards bibliographical research, none can show more valuable labour: unembarrassed by insular prejudices, assisted by that most valuable publication, the *Index Medicus*, no class of authors seeks more to do justice to all nations than that represented by the American medical profession." We can only congratulate our Western *confrères* on the fact that the value of their work is being now so well recognised wherever the English language is written.

*Physicians', Surgeons', and Consultants' Visiting List.*—Bristol: JOHN WRIGHT AND CO.—The objects in view in issuing another addition to the already numerous lists of this kind have been: 1, to economise space and provide the greatest amount of accommodation; 2, to economise the practitioner's time by enabling him to write his patients' names once a month only; 3, to include a consultant's record, giving at a glance the date and hour of appointment with the name of the medical man in attendance. We have carefully looked through this time-saving device: it has space for 160 cases per month, with special pages for consultation and many other memoranda. The book is of small bulk, and of convenient shape for the pocket. It deserves popularity.

*Temporal Welfare.* London: FRANCIS HODGSON.—This, which is described as "A Journal of Church Effort for the physical and social well-being of the people," is a new periodical, published in order to focus all work done by the Church for the temporal welfare of the people. It is based on the same lines as the Church of England Sanitary Association, and promises to assist by extending voluntary effort for the promotion of sanitary reform. In their constant struggle against ill-health, disease, and death, the members of the medical profession are glad of the assistance of such allies.

*The English Illustrated Magazine.*—This magazine, always in the forefront of good and popular literature, is not likely to depart from this position now that it has passed into the hands of the *Illustrated London News* management. The first two numbers of the new volume possess special interest for the medical reader. In that for October the arguments for and against tobacco smoking by women are well put by Lady Colin Campbell and Mrs. Lynn Linton. Medical opinion will probably come to say that there is nothing in the female organism which should forbid the use of tobacco by women more than by men. It is a question of custom and sentiment. Tobacco in moderation would doubtless be as useful to one sex as to the other. In the November number there is an appreciative notice of Surgeon-Major Parke.

